# Transgenic expression of dentin phosphoprotein (DPP) partially rescued the dentin defects of *DSPP*-null mice

**DOI:** 10.1371/journal.pone.0195854

**Published:** 2018-04-19

**Authors:** Hua Zhang, Xiaohua Xie, Peihong Liu, Tian Liang, Yongbo Lu, Chunlin Qin

**Affiliations:** 1 Department of Biomedical Sciences, Texas A&M University College of Dentistry, Dallas, TX, United States of America; 2 Department of Stomatology, the 2nd Affiliated Hospital of Harbin Medical University, Harbin, Heilongjiang, China; 3 Department of Periodontics, Harbin Medical University School of Stomatology, Harbin, Heilongjiang, China; University of Insubria, ITALY

## Abstract

Mutations in the dentin sialophosphoprotein (DSPP) gene cause dentinogenesis imperfecta. After synthesis, DSPP is proteolytically processed into NH_2_- and COOH-terminal fragments. The NH_2_-terminal fragment of DSPP is highly glycosylated but not phosphorylated, whereas the COOH-terminal fragment (named “dentin phosphoprotein” or “DPP”) is highly phosphorylated but not glycosylated. These two fragments are believed to perform distinct roles in dentin formation. To analyze the functions of DPP in dentinogenesis, we created “*Dspp*^-/-^;DPP Tg mice”, which expressed transgenic DPP driven by a Type I collagen promoter but lacked the endogenous *Dspp* gene. We characterized the dentin of the *Dspp*^-/-^;DPP Tg mice using X-ray radiography, histology, scanning electron microscopy, double fluorochrome labeling, immunohistochemistry and *in situ* hybridization. Micro-computed tomography analyses revealed that at postnatal 6 months, the transgenic expression of DPP increased the dentin thickness of the *Dspp*-null mice by 97.1% and restored the dentin material density by 29.5%. Histological analyses showed that the *Dspp*-null mice manifested an abnormal widening of the predentin while the predentin in *Dspp*^-/-^;DPP Tg mice was narrower than in the *Dspp*-null mice. Scanning electron microscopy analyses showed that the dentinal tubules in the *Dspp*^-/-^;DPP Tg mice were better organized than in the *Dspp*-null mice. The double fluorochrome labeling analyses demonstrated that the dentin mineral deposition rate in the *Dspp*^-/-^;DPP Tg mice was significantly improved compared to that in the *Dspp*-null mice. These findings indicate that the transgenic expression of DPP partially rescued the dentin defects of the *DSPP*-null mice, suggesting that DPP may promote dentin formation and that the coordinated actions between DPP and the NH_2_-terminal fragment of DSPP may be necessary for dentinogenesis.

## Introduction

Dentin is composed of an organic matrix that is primarily made of Type I collagen and a mineral phase that consists of hydroxyapatite crystals. During the formation of dentin, odontoblasts secrete a collagen-rich matrix termed “predentin”, which is not mineralized. As the precursor of dentin, predentin lies between the mineralization front and the cells; it is converted to dentin when hydroxyapatite crystals are laid down within and around collagen fibrils. During normal dentinogenesis, a rather uniform layer of predentin is maintained, suggesting that the rate of predentin formation must equal the rate of mineralization. The uniformity of these rates indicates that balanced mechanisms must be involved to control the site and rate of hydroxyapatite formation and growth. Likewise, in pathological conditions, the widening of the predentin layer must involve the incapacitation of a controlling mechanism. Dentinogenesis is a dynamic process involving multiple changes that occur within the window of time when predentin is formed and converted to dentin. Changes that are well documented include increases in collagen fibril diameters, secretion and removal of proteoglycans and deposition of acidic phosphoproteins at the mineralization front [1].

During dentinogenesis, Type I collagen, secreted by odontoblasts, forms the undergirding that is mineralized in a highly controlled manner. In addition to Type I collagen, the extracellular matrix (ECM) of dentin contains a number of non-collagenous proteins (NCPs) and proteoglycans. Dentin sialophosphoprotein (DSPP) belongs to the SIBLING (Small Integrin-Binding LIgand, N-linked Glycoprotein) family [2]. Excluding Type I collagen, DSPP and its processed fragments are the most abundant organic molecules in the dentin, accounting for more than 50% of the NCPs in the dentin ECM [3]. The importance of DSPP in dentinogenesis is supported by experiments showing the association of mutations in the *Dspp* gene with dentinogenesis imperfecta in humans [4, 5] and of the *Dspp* gene loss with dental defects in mice [6]. The predentin in the *Dspp* gene knockout mouse is hypomineralized (widening of predentin), giving rise to a phenotype similar to the manifestations of dentinogenesis imperfecta Type III in humans.

DSPP is a large protein that is proteolytically processed to form the COOH-terminal fragment and NH_2_-terminal fragment [3, 7]. The COOH-terminal fragment of DSPP is named “dentin phosphoprotein” (DPP), which is highly phosphorylated but not glycosylated [3]. The most unusual feature of DPP is the occurrence of large amounts of aspartic acid (Asp) and phosphoserine (Pse), mostly present in the repeating sequences of (Asp-Pse-Pse)_n_ and (Asp-Pse)_n_ [7]; this unique structure makes DPP the most polyanionic protein in mammals; DPP isolated from rat dentin has an isoelectric point of 1.1 [8, 9]. Computer-generated predictions portray these repeating sequences as extended backbone structures with relatively long ridges of carboxylate and phosphate groups on each side of the peptide backbone [10]. These structures fit well with the purported function of DPP in the nucleation and modulation of hydroxyapatite crystal formation since they bind multiple calcium ions in a highly oriented fashion. *In vitro* mineralization studies indicate that DPP is an important initiator and modulator for the formation and growth of hydroxyapatite crystals [11–13]. Other observations show that DPP is transported to the mineralization front following its synthesis and secretion by odontoblasts; here it binds to collagen fibrils and assumes a structure that promotes the formation of initial hydroxyapatite crystals. While this putative function for DPP in promoting mineralization is supported by some *in vitro* experiments [3, 14, 15], *in vivo* investigations are needed to further elucidate the biological functions of this unique phosphoprotein and test these beliefs.

The NH_2_-terminal fragment of DSPP encoded by the 5’ portion of the DSPP transcript exists in two forms: the core protein form known as “dentin sialoprotein” (DSP) [16] and the proteoglycan form referred to as “DSP-PG”, which has two glycosaminoglycan chains made of chondroitin sulfates [17]. DSP and DSP-PG have few or no phosphates [3], in clear contrast to the highly phosphorylated DPP that may have as many as 200 phosphates [3]. *In vitro* studies showed that DSP had little or no effect on the formation and growth of hydroxyapatite crystals [18] while there is a lack of information regarding the effects of DSP-PG on the formation and growth of the hydroxyapatite crystals.

The remarkable chemical differences between the COOH-terminal fragment (DPP) and NH_2_-terminal fragment (including DSP and DSP-PG) of DSPP suggest that these molecular variants may perform different functions in biomineralization although they are derived from the same mRNA. The abundance of DSPP fragments, along with the scarcity of full-length DSPP in the dentin, suggests that the processed fragments of DSPP may be the functional forms directly involved in dentin formation. Previously, we showed that the blocking of DSPP processing by substituting Asp^452^ with Ala^452^ at the cleavage site of mouse DSPP leads to the functional loss of this protein *in vivo*, indicating that the posttranslational cleavage to convert DSPP into fragments is an activation event, transforming an inactive precursor to active fragments that are essential to the formation and mineralization of dentin [19]. More recently, we showed that the transgenic expression of DSPP NH_2_-terminal fragment failed to rescue the dentin defects in *Dspp*-null mice [20]. In the present study, we created transgenic mice expressing DPP, and introduced the transgenic DPP into the *Dspp*-null background. We observed that the transgenic expression of DPP partially rescued the dentin defects of *Dspp*-null mice.

## Materials and methods

### Generation of mice expressing transgenic DPP in the *Dspp*-null background

The generation of transgenic mice expressing DPP under the control of a 3.6 kb Type I collagen (Col1a1) promoter has been described in our previous publication [21]. The transgene (referred to as “Col1a1-HA-DPP”) contains the sequence encoding the hemagglutinin (HA)-tag at the amino-terminal end of the DPP, which allows the detection of the transgenic protein by the anti-HA antibodies [21]. Transgenic mice were identified by PCR analyses of genomic DNA extracted from tail biopsy tissues using the following primers specific for the transgene: forward primer, 5’-TGGTGGTGCAAATCAAAGAA-3’, located on the DPP coding sequence, and reverse primer, 5’-CTGTCACTGTCACCATCACCATTAC-3’, located on the SV40 polyadenylation signal. While we obtained a total of fifteen Col1a1-HA-DPP founder mice (15 lines) from multiple pronuclear injections [21], only one mouse line (line 2) can be passed to F3 and subsequent generations (21). The other 14 founder mice either died within 6 months after birth, or failed to pass the transgene on to their offspring, or could not produce pups beyond F1. In this investigation, the transgenic line 2 (DPP Tg2) mice at F3 and subsequent generations were crossed with *Dspp* ^-/-^ mice (strain name: B6; 129-Dspp^tm1Kul^/Mmnc; MMRRC, UNC, Chapel Hill, NC, USA) to introduce the Col1a1-HA-DPP transgene into the *Dspp*-null background (lacking the endogenous *Dspp* gene). The mice expressing the Col1a1-HA-DPP transgene without the endogenous *Dspp* gene were referred to as *Dspp*^-/-^;DPP Tg mice. The primers used to identify the endogenous *Dspp* alleles and null alleles (containing the *LacZ* gene) were described in our previous report [19]. The age- and gender-matched littermate *Dspp* heterozygous (*Dspp*^+/-^) mice were used as normal control mice since there was no significant phenotypic difference between the *Dspp*^+/-^ and wild-type (*Dspp*^+/+^) mice as we and others have previously reported [6, 19, 20]. We analyzed the teeth of *Dspp*^-/-^;DPP Tg mice, in comparison with those from the *Dspp*^+/-^ and *Dspp*^-/-^ mice to determine the effects of transgenic HA-DPP on the formation and mineralization of the dentin in the *Dspp*-null molars.

All the animals used in the present study were housed in a temperature-controlled vivarium on a 12–12 light cycle with free access to food and water. All animal procedures were performed in accordance with the National Institutes of Health Guide for the Care and Use of Laboratory Animals and approved by the Institutional Animal Care and Use Committee of Texas A&M University College of Dentistry (Dallas, TX, USA).

### X-ray radiography

The mandibles dissected from the *Dspp*^+/-^, *Dspp*^-/-^, and *Dspp-/-;DPP Tg* mice at 3 and 6 months of age were examined by both plain X-ray radiography (Faxitron MX-20DC12 system; Faxitron Bioptics, Tucson, Arizona 85706 USA Tucson, AZ, USATucson, Arizona 85706 USA) and microcomputed tomography (μCT) system (Scanco μCT35 imaging system; Scanco Medical, Brüttisellen, Switzerland). For the μCT analyses, a low-resolution scan (20 μm slice increment) was performed for an overall morphological assessment of the molar regions in the mandibles. A high-resolution scan in 3.5 μm slice increments was performed on 6-month-old samples to examine the mandibular first molars. A morphometric parameter analysis was performed using the built-in μCT software system. The dentin thickness, dentin volume (DV), total tissue volume (TV), DV/TV, and dentin material density of the mandibular first molars were obtained at a threshold of 375. The data acquired from the high-resolution scans of four samples per group were used for the quantitative analysis.

### Histology, immunohistochemistry, *in situ* hybridization and picro-sirius red staining

For histologic analysis, the mandibles were fixed in freshly prepared 4% paraformaldehyde in phosphate-buffered saline (pH 7.4) at 4°C overnight and then decalcified in 15% ethylenediaminetetraacetate (EDTA) solution (pH 7.4) at 4°C for 5~14 days depending on the ages of the animals. The samples were embedded in paraffin using standard histological procedures. Serial sections were cut at a thickness of 5 μm and used for Hematoxylin and Eosin (H&E) staining, immunohistochemistry (IHC), *in situ* hybridization, or picro-sirius red staining.

For IHC analyses, the experiments were carried out using an ABC kit, a M.O.M. kit and a DAB kit (Vector Laboratories, Burlingame, CA) according to the manufacturer’s instructions. We employed polyclonal antibodies against DSP (the NH_2_-terminal fragment of DSPP) [16] at a dilution of 1:800 and biglycan (LF-159, a gift from Dr. Larry Fisher of the Craniofacial and Skeletal Disease Branch, National Institutes of Health, Bethesda, MA, USA) at a dilution of 1:1000 to detect DSP and biglycan. Monoclonal anti-HA antibody (Covance Inc., Dallas, TX, USA) at a dilution of 1:2000 was used to reveal the HA epitope tagged to DPP protein. The sections were counterstained with methyl green. The same concentrations of normal mouse IgG or rabbit IgG were used to replace the monoclonal or polyclonal antibodies to serve as negative controls.

*In situ* hybridization was carried out to assess the mRNA level of Type I collagen (Col 1a1) in the mandibular molars of 3-week-old mice as described previously [22]. The RNA probes were labeled with digoxigenin (DIG) using a RNA Labeling Kit (Roche, Indianapolis, IN) according to the manufacturer’s instruction. DIG-labeled RNA probes were detected by an enzyme-linked immunoassay with a specific anti-DIG-alkaline phosphatase antibody conjugate (Roche, Indianapolis, IN) and a VECTOR red alkaline phosphatase substrate (Vector Laboratories, Burlingame, CA), which produced a red color for positive signals. The sections were counterstained with methyl green.

For picro-sirius red staining, the mandible sections were immersed in haematoxylin solution for 8 minutes to stain the nuclei and washed for 10 minutes in water. Then, the sections were stained in picro-sirius red for one hour, washed in two changes of acidified water, dehydrated in three changes of 100% ethanol, cleared in xylene and mounted. The structure and organization of collagen fibers in the dentin were examined under a polarized light microscope.

Microphotographs were taking under an Olympus BX51 microscope equipped with an Olympus DP72 microscope digital camera interfaced with Olympus cellSens Standard 1.6 software (Olympus America Inc., Center Valley, PA).

### Double fluorochrome labeling of dentin

Double fluorescence labeling was performed to analyze the mineral deposition in the dentin in the mouse incisors, as we described previously [19, 23]. Briefly, calcein (green) (Sigma Aldrich, St. Louis, MO) at a 10 mg/kg concentration was administered intraperitoneally to the 5-week-old mice. Alizarin red (Sigma Aldrich) at a 30 mg/kg concentration was injected intraperitoneally 1 week later. The mice were sacrificed 48 hours after the alizarin red was injected. The mandibles were then dissected and fixed in 4% paraformaldehyde in 0.1 M phosphate buffer solution (pH 7.4) at 4°C for 48 hours. The tissue specimens were dehydrated in ascending concentrations of ethanol (from 70% to 100%) and then embedded in methyl methacrylate (MMA, Buehler, Lake Bluff, Illinois, USA) without decalcification. Sections (10 μm thick) were prepared and the incisor dentin region under the mesial root of the first molar was viewed under epifluorescent illumination with a Nikon E800 microscope interfaced with Osteomeasure histomorphometry software (version 4.1, Atlanta, GA). The distances between the green and red zones were measured and calculated to estimate the calcium deposition rate. Three mice from each group were used for quantitative analysis.

### Resin-casted backscattered and acid-etched scanning electron microscopy (SEM)

For the SEM analyses, the mandibles from 3-month-old mice were dissected and fixed with 4% paraformaldehyde in 0.1 M phosphate buffer solution (pH 7.4) at 4°C for 48 hours. MMA-embedded specimens were prepared as described above. After adjusting a suitable comparable position of the samples, sand paper was used to grind the acrylic block with an increasing order of grit fineness. These samples were then polished using micro cloth with Metadi Supreme Polycrystalline diamond suspensions of sizes 0.1 micron, 0.25 micron and 0.05 micron (Buehler). These samples were then washed using the ultrasonic washer and placed in the vacuum system for 2 days. The dehydrated specimens were sputter-coated with carbon and scanned with a backscattered electron detector in a FEI/Philips XL30 Field emission environmental SEM system (JSM-6010LA, JEOL, Japan). To assess the dentinal tubule structures, the carbon-coated samples were repolished, as described above. The surfaces were acid-etched with 12% phosphoric acid for 7 seconds, washed with 5% sodium hypochlorite for 35 minutes, coated with gold and palladium, and then examined by SEM.

### Statistical analysis

The data analysis was performed with a one-way analysis of variance ANOVA for multiple-group comparison. If significant differences were found with the one-way ANOVA, the Tukey-Kramer post-hoc test was used to determine which groups were significantly different from others. The data were expressed in terms of mean ± SD. (standard deviation). P<0.05 was considered as the statistical significant difference.

## Results

### Transgenic expression of Col1a1-HA-DPP in the teeth

**Fig 1:**
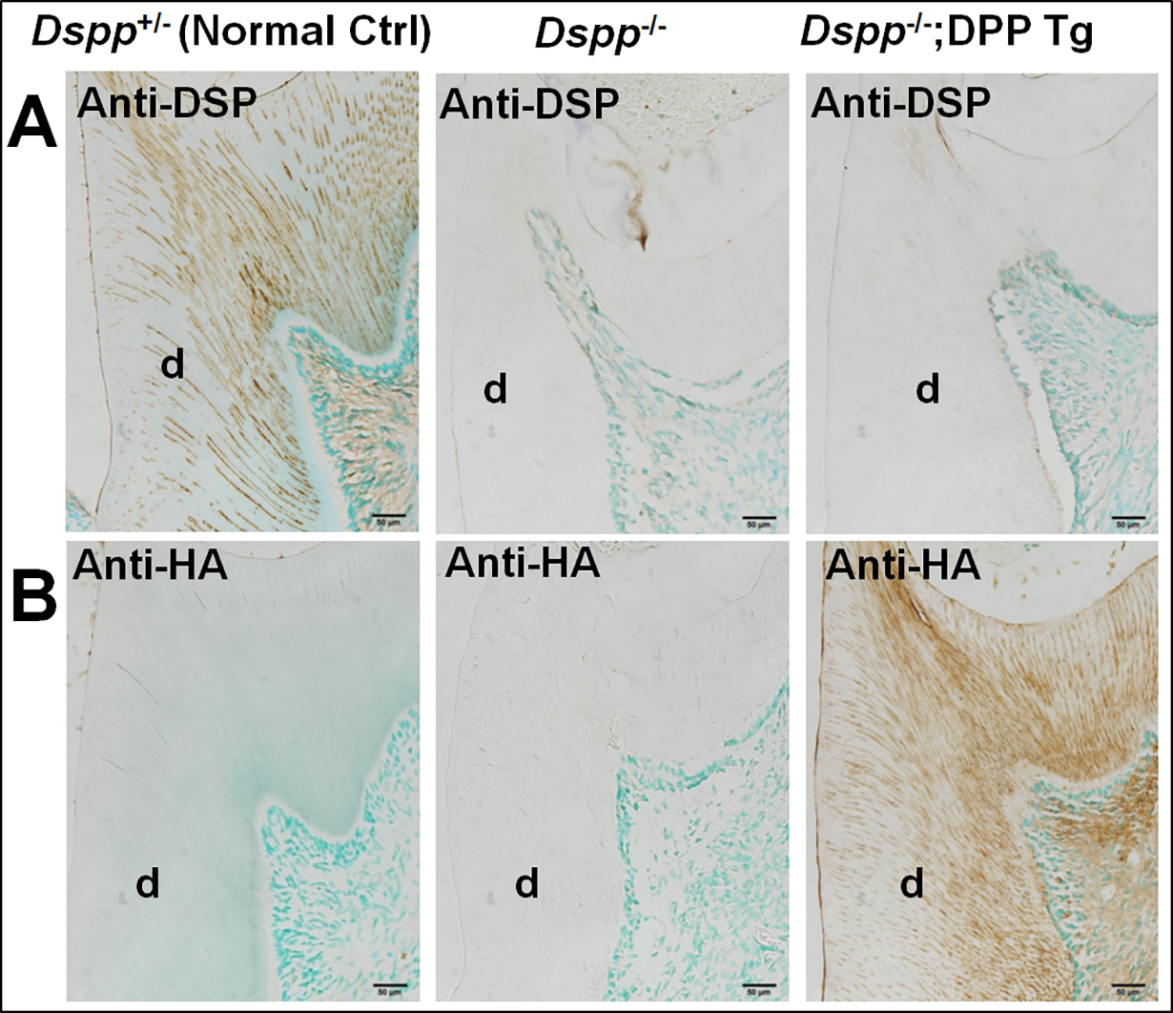
Immunohistochemical detection of DSP and Col1a1-HA-DPP in the mandibular first molars of 3-month-old *Dspp*^*+/-*^, *Dspp*^*-/-*^ and *Dspp*^*-/-*^;DPP Tg mice. Immunohistochemical staining showed the presence of the anti-DSP signals (brown color) in the dentin matrix (d) of the *Dspp*^*+/-*^ mice (A, left image), but not in the *Dspp*^*-/-*^ (A, middle image) and *Dspp*^*-/-*^;DPP Tg mice (A, right image). Note the presence of HA-DPP (Anti-HA) signals in the *Dspp*^*-/-*^;DPP Tg mice (B, right image). Scale bars = 50 μm.

IHC analyses using the anti-DSP polyclonal antibody revealed the presence of this protein (i.e., the NH_2_-terminal fragment of DSPP) in the *Dspp*^+/-^ (normal control) mice and its absence in the *Dspp*^-/-^ and *Dspp* ^-/-^;DPP Tg mice (Fig 1). In the *Dspp*^+/-^ mice, DSP was found in the dentin matrix, odontoblasts and dental pulp. IHC analyses using an anti-HA monoclonal antibody confirmed the expression of the transgene in the dentin-pulp complex; the HA-DPP protein signals were distributed in the dentin matrices, the odontoblasts and dental pulp, demonstrating a distribution pattern similar to that of DSP (Fig 1).

X-ray radiography showed that the mandibular teeth from 3-week-old *Dspp*^+/+^;DPP Tg transgenic mice were slightly smaller than the normal controls, and H&E staining of the mandibular first molars revealed that the dentin of *Dspp*^+/+^;DPP Tg mice appeared slightly thinner than in the normal control mice (data not shown).

### Transgenic expression of Col1a1-HA-DPP partially rescued the dentin defects of *Dspp*^-/-^ mice

**Fig 2:**
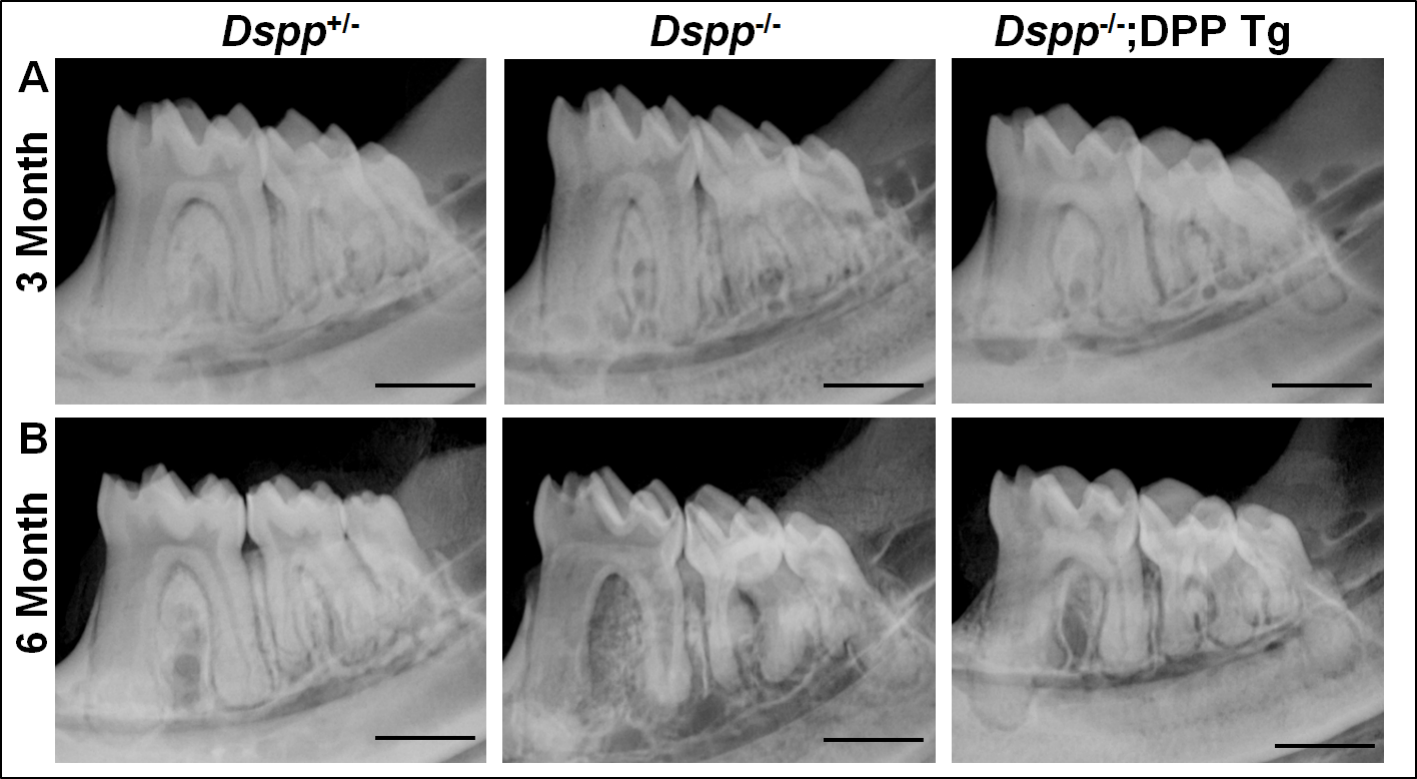
Plain X-ray radiographs of mandibular molars from 3- and 6-month-old mice. The mandibular molars in the *Dspp*^-/-^ mice had an enlarged pulp chamber and thinner dentin compared with the *Dspp*^+/-^ mice; the teeth in the *Dspp*^*-/-*^;DPP Tg mice appeared to be similar to those of the *Dspp*^+/-^ mice. Scale bars in A and B = 1 mm.

**Fig 3:**
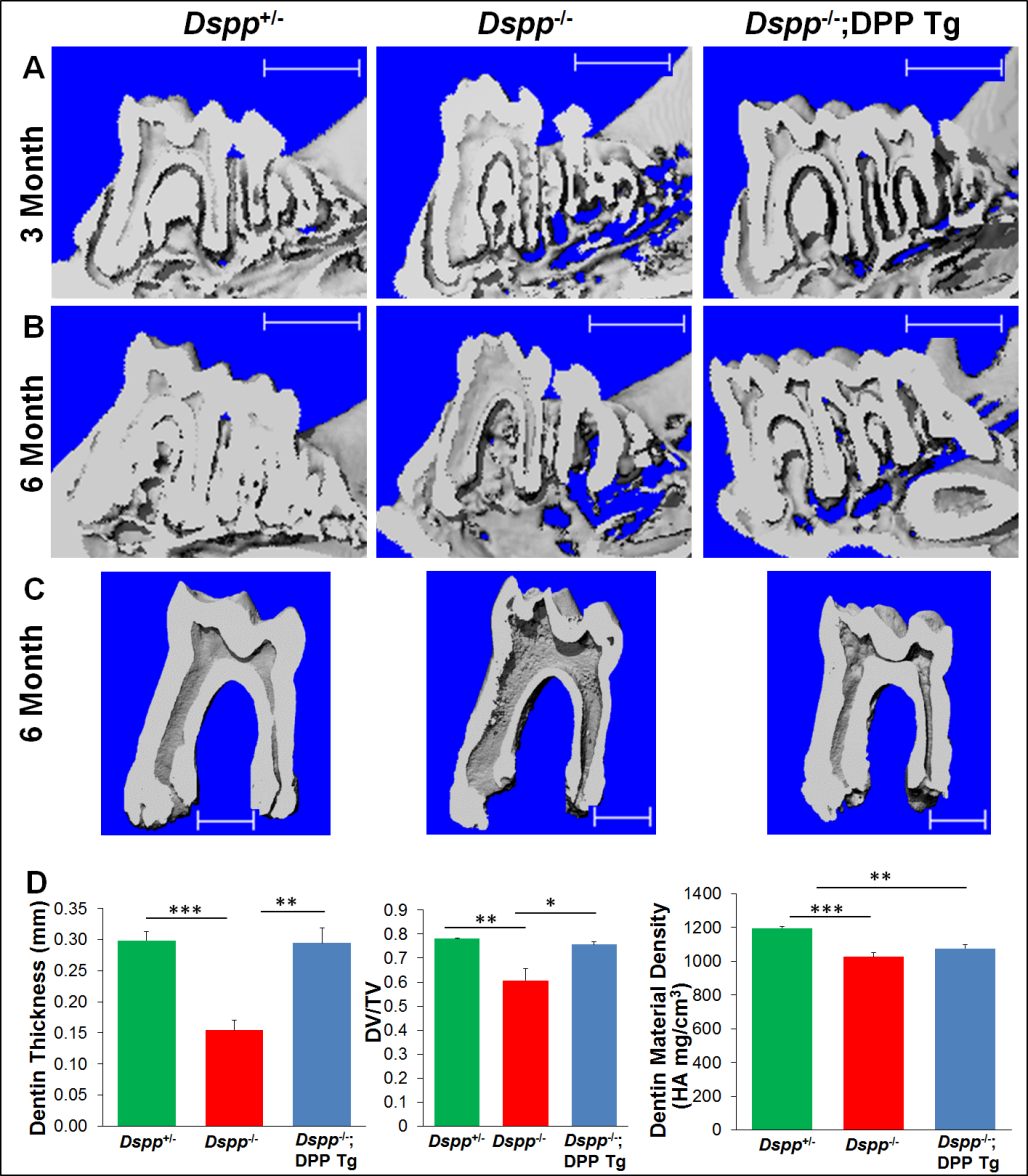
μCT analyses of the mandibles and molars from 3- and 6-month-old mice. A and B are representative μCT images of the mandibles from 3- and 6-month-old *Dspp*^+/-^, *Dspp*^-/-^, and *Dspp-/-;DPP Tg* mice. C are the 3D reconstruction images of the mandibular first molars. The quantitative μCT analysis (D) demonstrated significant correction of dentin thickness and dentin volume in *Dspp-/-;DPP Tg* mice compared to the *Dspp*^+/-^ and *Dspp*^-/-^ mice. Values of dentin volume are expressed as a ratio of dentin volume (DV) to total tissue volume (TV), where the TV is a sum of the dentin and pulp volume. Dentin mineral density analyses show a slight improvement of dentin mineralization in *Dspp-/-;DPP Tg* mice. Data in D are presented as the mean ± SD (n = 4). *: p<0.05, **: p<0.01, ***: p<0.001. Scale bars in A and B = 1.0 mm; scale bars in C = 500 μm.

We first performed plain X-ray radiography analyses to assess the tooth structure and morphology. Plain X-ray radiography revealed that the *Dspp*^-/-^ mice exhibited thinner dentin and enlarged pulp chambers compared with the *Dspp*^+/-^ mice, and the transgenic expression of HA-DPP markedly improved the dental defects of the *Dspp*^-/-^ mice (Fig 2). The microcomputed tomography (μCT) analyses further confirmed that the transgenic expression of HA-DPP significantly corrected the dentin defects of the *Dspp*^-/-^ mice (Fig 3). The quantitative μCT analyses (Fig 3D) showed that the expression of the HA-DPP transgene restored the dentin thickness (n = 4, p<0.01) and dentin volume (n = 4, p<0.05) of the mandibular first molars in the *Dspp*^-/-^ mice to the level of the *Dspp*^+/-^ (normal control) mice at 6-months of age. However, the transgenic DPP expression only slightly improved the dentin material density (n = 4, p>0.05) of the *Dspp*^-/-^ mice.

**Fig 4:**
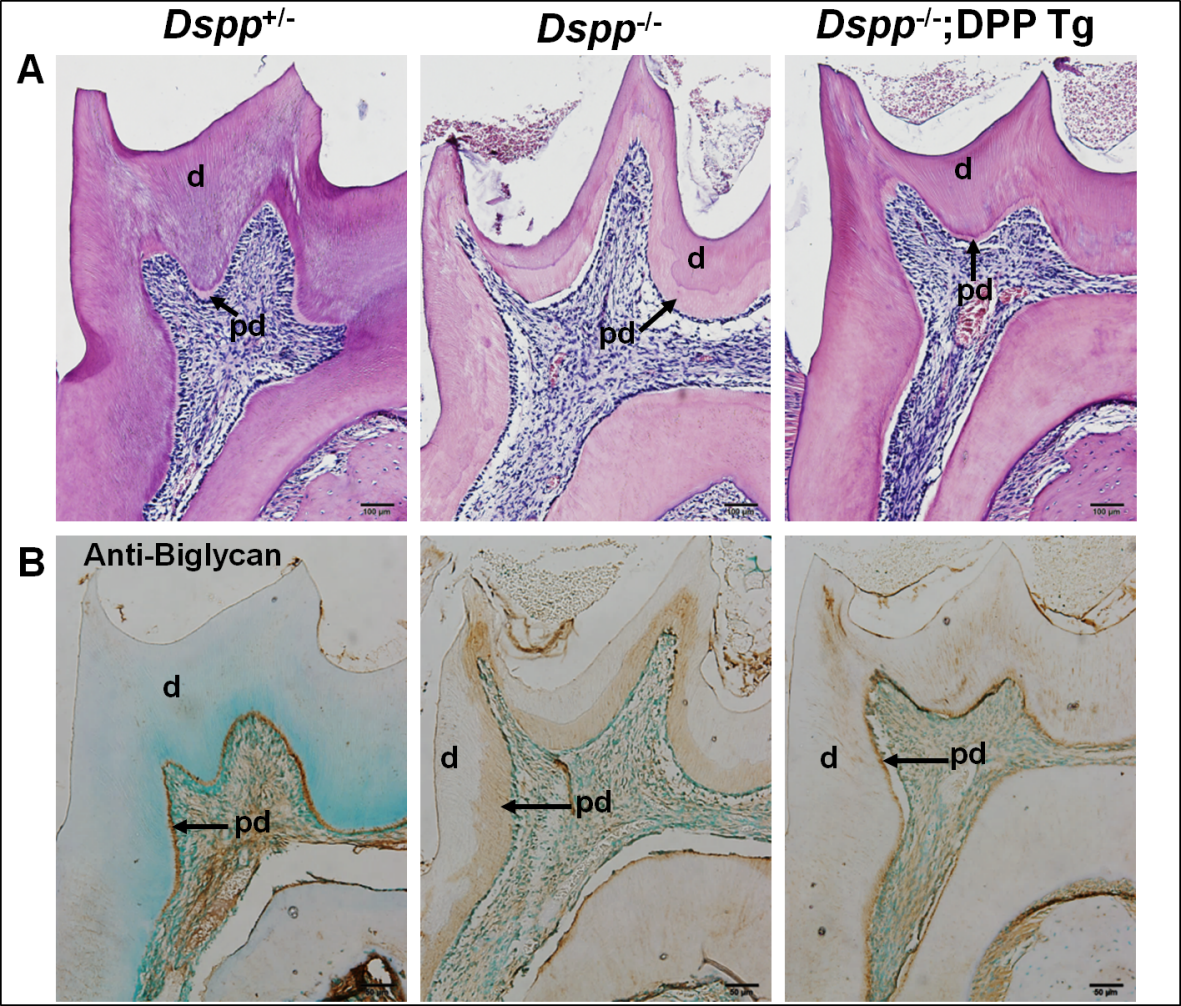
H&E staining and anti-biglycan immunostaining of the mandibular first molars from 3-month old mice. H&E staining (A) showed that the reduced dentin thickness, widened predentin zone, irregular dentin-predentin border and enlarged pulp chambers in the *Dspp*^-/-^ mice were corrected in the *Dspp-/-;DPP Tg* mice. The anti-biglycan immunostaining (B) revealed that the width of the predentin zone in the *Dspp-/-;DPP Tg* mice was similar to that of the *Dspp* ^+/-^ mice. d, dentin; pd, predentin. Scale bars = 50 μm in A and B.

H&E staining analyses (Fig 4A) further validated the rescue effects of HA-DPP on the dentin defects of the *Dspp*^-/-^ mice. At the age of 3 months, the mandibular molars of the *Dspp*^-/-^ mice displayed enlarged pulp chambers, reduced dentin thickness, and a widened predentin zone compared with those of the *Dspp*^+/-^ mice; the structure of the dentin-pulp complex in the *Dspp* ^-/-^;DPP Tg mice appeared to be similar to that of the *Dspp*^+/-^ mice. Biglycan is an odontoblast terminal differentiation marker and is primarily localized in the predentin zone of the teeth [24]. The immunohistochemical staining revealed that the *Dspp*^-/-^ mice had a widened zone of biglycan staining signals, whereas the *Dspp* ^-/-^;DPP Tg mice displayed a biglycan distribution pattern similar to the *Dspp* ^+/-^ mice, suggesting that the widened predentin zone in the *Dspp* ^-/-^;DPP Tg mice was restored to a level similar to that in the *Dspp* ^+/-^ mice (Fig 4B). Taken together, these data showed that the transgenic expression of HA-DPP partially rescued the dentin defects of the *Dspp* ^-/-^ mice.

### Transgenic expression of HA-DPP partially rescued the dentin deposition rate in *Dspp*^-/-^ mice

**Fig 5:**
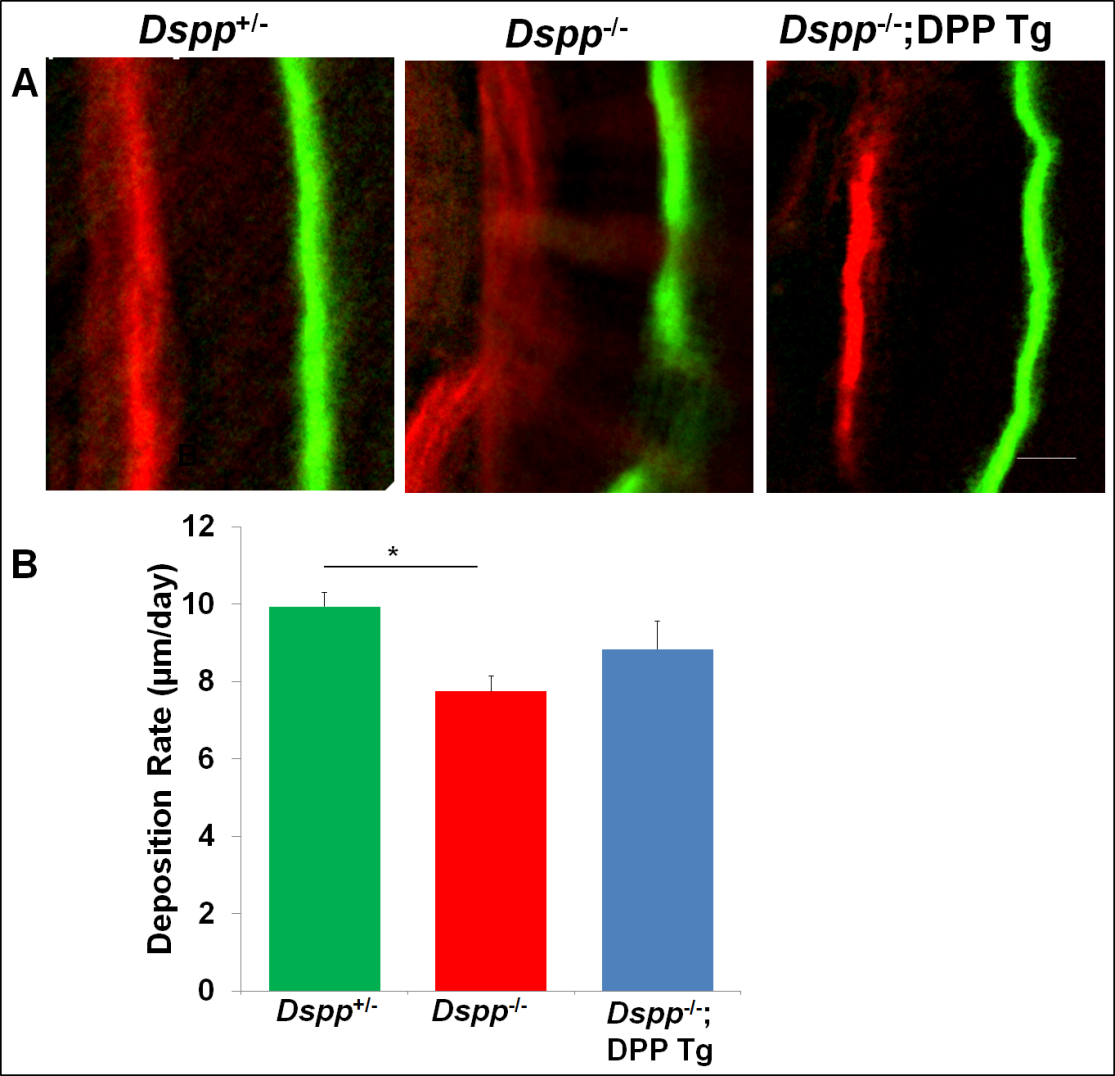
Double fluorochrome labeling to assess the mineral deposition in the incisor dentin. A are the representative images taken from the incisor dentin in the region under the mesial root of the mandibular first molars. The distance between the red- and green-labeled lines represented the dentin matrix mineralized over a period of 7 days. Compared to the *Dspp*^+/-^ mice, the labeled lines in the *Dspp*^-/-^ mice were thicker and diffused. The labeled lines in *Dspp-/-;*DPP Tg mice showed were uniform, and similar to those of *Dspp*^+/-^ mice. Scale bars = 20 μm. The quantitative analyses (B) showed that the dentin of the *Dspp*^-/-^ mice had a lower mineral deposition rate compared with the *Dspp*^+/-^ mice, and the transgenic expression of DPP improved the dentin mineral deposition rate of the *Dspp*^-/-^ mice. Data are presented as the mean ± SD (n = 3). *p<0.05.

The double fluorochrome labeling analysis (Fig 5) showed two sharp and well-demarcated fluorescent label lines in either the *Dspp* ^+/-^ or *Dspp* ^-/-^;DPP Tg mice, whereas the two label lines were blurry and diffused in the *Dspp* ^-/-^ mice. The quantitative analyses indicated that the expression of transgenic DPP improved the dentin mineral deposition rate of the *Dspp*^-/-^ mice.

### Transgenic expression of HA-DPP alleviated the hypomineralization defects and partially corrected the dentinal tubule abnormalities in the dentin of *Dspp*^-/-^ mice

**Fig 6:**
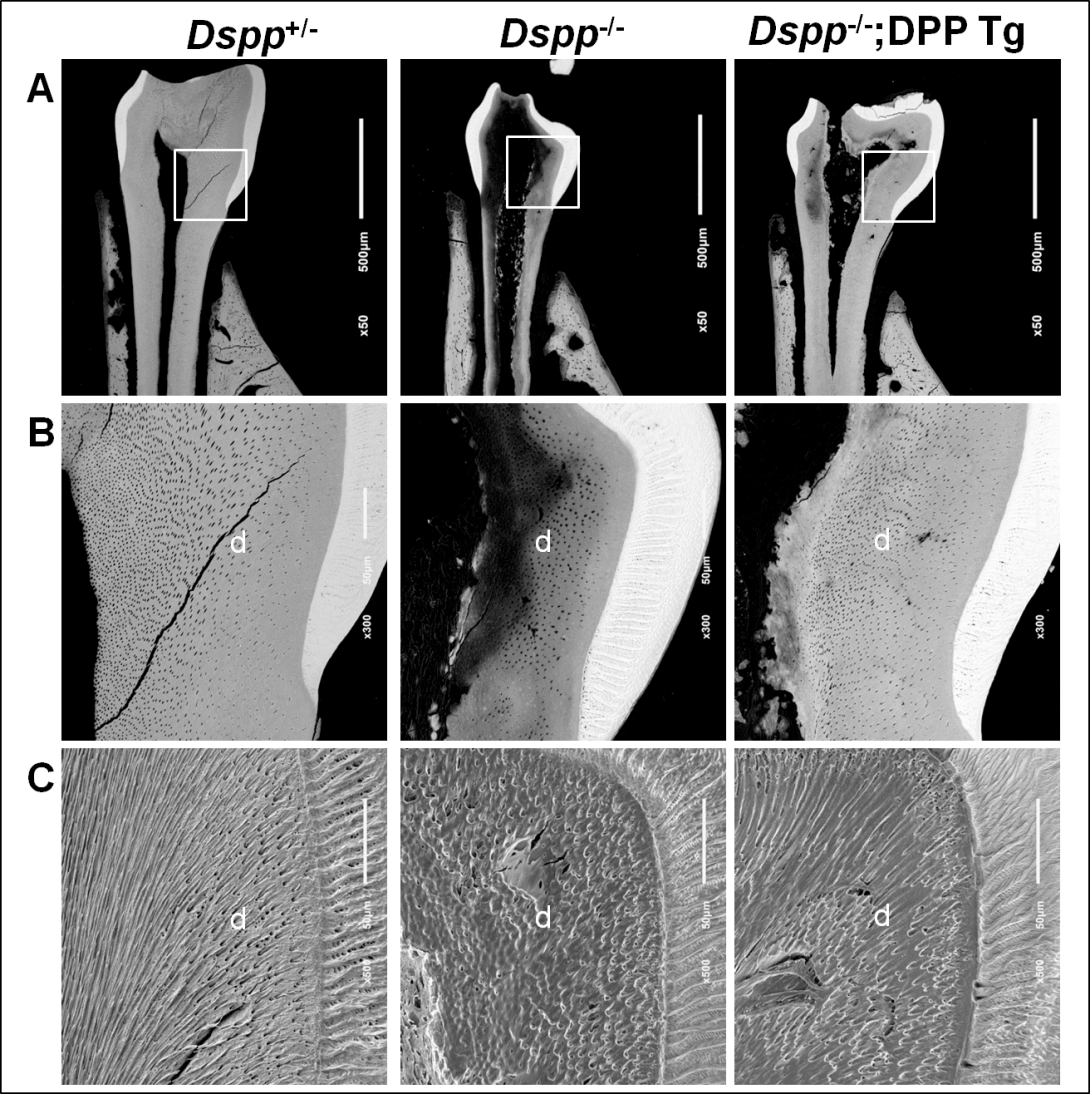
Backscattered SEM and resin-casted and acid-etched SEM analyses of the mandibular first molars from 3-month-old mice. Representative backscattered SEM images (A) showed enamel in white (higher level of mineralization), dentin in gray, and areas of less or no minerals in black. The dentin in the *Dspp*^-/-^ was thinner, had more black areas, with irregular mineralization front, while the dentin thickness and mineralization in the *Dspp*^*-/-*^;DPP Tg mice was greatly improved. The images in B are enlarged views of the boxed areas in A. Scale bars: A, 500 μm, B, 50 μm. Representative resin-casted and acid-etched SEM images (C) revealed that the dentinal tubular structures were poorly formed in the *Dspp*^-/-^ mice. Although not fully rescued, the dentinal tubules in the *Dspp*^*-/-*^;DPP Tg mice were markedly improved, compared to those in the *Dspp*^-/-^ mice. Scale bars = 50 μm.

Backscattered and acid-etched SEM (scanning electron microscopy) analyses were carried out to analyze the effects of the HA-DPP transgene on the dentin mineralization defects and the dentinal tubular structural abnormalities in the *Dspp* ^+/-^ mice. The backscattered SEM analysis demonstrated that the *Dspp* ^-/-^ mice displayed large areas of unmineralized or hypomineralized dentin (black areas) compared with the *Dspp* ^+/-^ mice (Fig 6A and 6B). The transgenic expression of HA-DPP remarkably reduced the areas of the hypomineralized dentin and increased the dentin thickness in the *Dspp* ^-/-^;DPP Tg mice (Fig 6A and 6B). The acid-etched SEM analysis images revealed that the dentinal tubules in the *Dspp*^+/-^ mice were well organized and evenly distributed, whereas those in the *Dspp* ^-/-^ mice were poorly formed and disorganized (Fig 6C). The HA-DPP transgene dramatically improved the structure and organization of the dentinal tubules in the *Dspp*^*-/-*^;DPP Tg (Fig 6C).

### The effect of transgenic HA-DPP on the collagen expression in the odontoblasts and collagen organization in the dentin matrix

**Fig 7:**
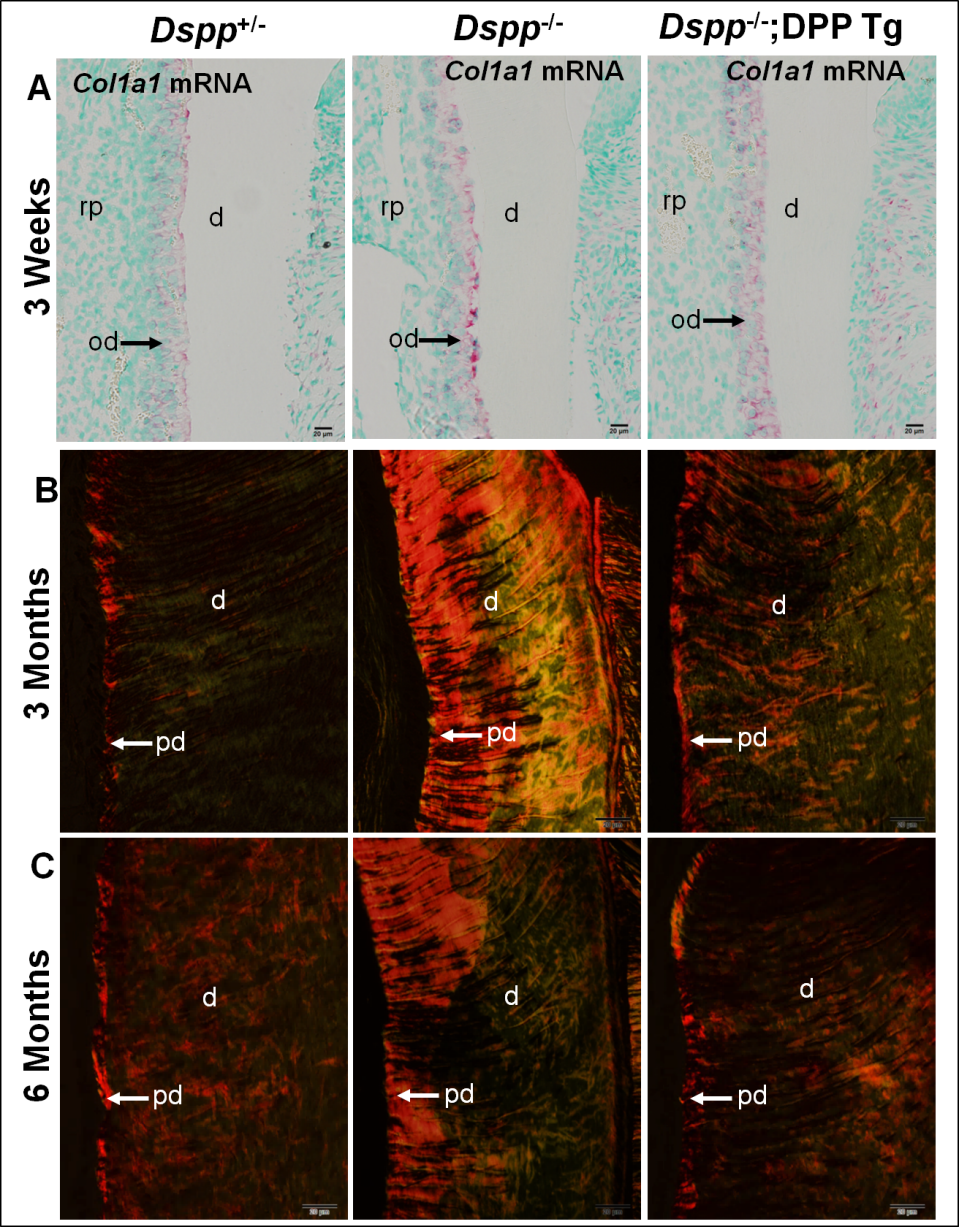
The effect of transgenic DPP on the collagen expression and organization in the *Dspp*-null mice. *In situ* hybridization analyses of Col Ia1 mRNA in the mandibular first molars of 3-week-old mice indicated that there was no apparent difference in the signal intensities (pink/red color) in the odontoblasts among the three groups (A). Picro-sirius Red-staining showed that a great amount of larger-diameter collagen fibers were found in the predentin and dentin, and were disorganized in the *Dspp*^-/-^ mice, whereas only a small amount of larger-diameter collagen fibers were restricted to the predentin in the *Dspp*^+/-^ mice and *Dspp-/-;DPP Tg* mice. d, dentin; rp, radiocular pulp; pd, predentin; and od, odontoblast. Scale bars = 20 μm.

*In situ* hybridization analyses showed that there was no apparent difference in the expression level of the *Col1a1* gene in the odontoblasts among these three groups of mice (Fig 7A). When the Picro-sirius Red stained sections are observed under polarized light microscope, the larger collagen fibers are shown in bright yellow or orange color, whereas thinner collagen fibers, including reticular fibers are in green. Picro-sirius red staining of the mandibular first molars from 3- and 6-month-old mice revealed that in the *Dspp*^+/-^ mice, the larger collagen fibers were mainly found in the predentin zone, whereas the thinner collagen fibers were evenly distributed in the dentin matrix (Fig 7B and 7C). In the *Dspp*^-/-^ mice, however, larger collagen fibers were abundantly present in both the predentin and dentin matrices and were irregularly organized (Fig 7B and 7C). In the *Dspp*^*-/-*^;DPP Tg mice, the distribution of the collagen fibers was very similar to that of the *Dspp*^+/-^ mice (Fig 7B and 7C). These findings suggested that while DPP does not affect the expression of *Col1a1*, but it might directly or indirectly influence the organization of collagen fibrils and the conversion of larger collagen fibers into thinner ones.

## Discussion

While the fact that the inactivation or loss of DSPP causes dentin defects has clearly illustrated its critical role in dentinogenesis, the exact mechanisms of how this most prominent dentin NCP controls the formation and mineralization of dentin are unclear. DSPP is cleaved into NH_2_-fragment and COOH-terminal fragment; the chemical structures of the former are dramatically different from the latter, suggesting potential differences in their functions [3]. While *in vitro* studies showed that the COOH-terminal fragment of DSPP (i.e., DPP) promotes the formation and growth of the hydroxyapatite crystals [11–13], there has been a lack of convincing *in vivo* data to elucidate the physiological functions of DPP in dentinogenesis. The findings that the introduction of transgenic DPP significantly improved the dentin formation in the *Dspp*-null mice confirmed the mineralization-promoting effects of DPP, consistent with the data in previous *in vitro* analyses.

The current study showed that the transgenic expression of DPP partially rescued the dentin defects in the *Dspp*-null mice. Previously, we demonstrated that the transgenic expression of the full-length normal DSPP driven by the same Type I collagen promoter completely rescued the dentin defects in the *Dspp*-null mice. The partial rescue of *Dspp*-deficient dentin by transgenic DPP, rather than the full restoration of *Dspp*-deficient dentin abnormalities, could be attributed to the following two reasons: 1) The proper function of DPP may require the assistance of the DSPP NH_2_-terminal fragment; the latter may be necessary for proper packaging/folding of the former in the endoplasmic reticulum and Golgi apparatus and for the subsequent delivery of DPP to the correct site. DSPP is cleaved into the NH_2_- and COOH-terminal fragments by bone morphogenetic protein 1/tolloid-like metalloproteinases [25, 26]. The metalloproteinases are initially synthesized as inactive precursors and become activated in the trans-Golgi network [27]. We envision that after translation, DSPP enters the endoplasmic reticulum and is delivered to the cis-Golgi network in its full-length form. In the endoplasmic reticulum and Golgi network, the NH_2_-terminal fragment of DSPP and other molecules serving as chaperons may assist in the proper folding of DPP. In the trans-Golgi network, DSPP is cleaved and activated into its functional forms by bone morphogenetic protein 1/tolloid-like metalloproteinases. 2) The second reason for the partial rescue may be due to the fact that the expression level of the transgenic DPP was not high enough. As the transgenic founders expressing high levels of DPP could not be passed to subsequent generations, we could not perform rescuing experiments with these mouse lines expressing higher levels of the transgene [21].

The transmission electron microscopy studies showed that DPP binds to Type I collagen predominantly at the "e" band in the gap region [28]. The binding interaction between DPP and Type I collagen induced a local conformational change in the collagen, bending the molecule and reducing its effective length [29]. While mineralization is believed to occur as the regulatory proteins such as DPP are deposited on the preformed fibrils [30], we postulate that dentinogenesis is a dynamic and reciprocal process. The proper assembly of Type I collagen is a prerequisite for the correct deposition of hydroxyapatite crystals into the collagen scaffold, and vice versa, appropriate mineralization promotes the correct assembly of Type I collagen during dentinogenesis. The fact that the transgenic expression of DPP improved the collagen structure of the dentin matrix indicates an important role of DPP in the formation of the correct collagen fibrils and lends support to our belief regarding the reciprocal relationships between collagen assembly and mineral deposition.

In summary, this study demonstrated that the transgenic expression of DPP significantly improved the dentin defects in *Dspp*-null mice. These data provide *in vivo* evidence that DPP may promote the deposition of hydroxyapatite crystals during the formation and mineralization of dentin, which is in agreement with the *in vitro* findings described in earlier reports. The partial restoration of *Dspp*-deficient dentin by transgenic DPP, rather than a full rescue, suggests that the balanced actions between the COOH- and the NH_2_-terminal fragments of DSPP may be required for dentin development.
